# Coexistence of gastric cancer and gastric GIST with intra-tumor bleeding: successful embolization with subsequent total gastrectomy

**DOI:** 10.1186/s40792-021-01244-1

**Published:** 2021-07-09

**Authors:** Raisuke Nishiyama, Toshihito Ogasawara, Nana Mamuro, Yutarou Kamei, Misuzu Yamada, Daisuke Furukawa, Toshiyuki Suzuki, Takayuki Nishi, Hideo Shimada

**Affiliations:** 1 Department of Emergency and Critical Care Medicine, Tokai University School of Medicine, Oiso Hospital, 21-1 Gakkyo, Oiso, Naka-Gun Kanagawa 259-1198 Japan; 2 Department of Surgery, Tokai University School of Medicine, Oiso Hospital, 21-1 Gakkyo, Oiso, Naka-Gun Kanagawa 259-1198 Japan

**Keywords:** Gastric cancer, Gastrointestinal stromal tumor (GIST), Transcatheter arterial embolization (TAE)

## Abstract

**Background:**

Gastrointestinal stromal tumor (GIST) is a rare tumor, however, simultaneous development of gastric cancer and gastric GIST has been documented more frequently in recent years. Rupture of gastric GIST is even more rare and occurred in 7% of all GISTs. Although ruptured GIST might be occasionally difficult to be managed by endoscopy, transcatheter arterial embolization (TAE) was reported to control bleeding from GIST effectively. We report herein a case of coexistence of gastric cancer and gastric GIST with progressing intra-tumor bleeding managed successfully by TAE and review the clinicopathological characteristics of this rare condition reported previously in the Japanese literature.

**Case presentation:**

A 75-year-old woman with dyspnea and systemic edema was diagnosed as simultaneous occurrence of gastric cancer (histopathologically detected tubular adenocarcinoma pT2N1M0 fStageIIA) and gastric GIST (65 × 92 mm in diameter at the anterior wall of the fornix) with intra-tumor hemorrhage. Perceiving the progress of bleeding from tumor growth and exacerbating anemia, TAE of left gastric artery was performed. Then remission of anemia has been obtained, the patient underwent an elective radical surgery.

**Conclusions:**

Simultaneous occurrence of gastric cancer and gastric GIST was speculated to be more common. TAE for ruptured GIST may be effective for hemostasis and reduction of tumor burden, which could facilitate minimal invasive surgery.

## Background

Gastrointestinal stromal tumor (GIST) is a rare tumor with an estimated incidence of 1–2/1,00,000/year [1], however, simultaneous development of gastric cancer and gastric GIST has been addressed in literature. Half of the coexisting GISTs were detected incidentally during or after surgery for gastric cancer. GIST becomes clinically apparent with broad spectrum of symptoms, ranging from unspecific abdominal discomfort to life-threatening gastrointestinal hemorrhage. Rupture of GIST is rare and occurred in 7% of all GISTs [2], however, frequency of intra-tumor bleeding, presumed to be in pre-rupture state, is unclear. Although endoscopy is the first choice for detecting and treating gastrointestinal hemorrhage, ruptured GIST might be difficult to be managed by endoscopy. And the emergency surgery has been performed occasionally despite significant morbidity and mortality. If endoscopic intervention fails, transcatheter arterial embolization (TAE) was reported to control bleeding effectively for ruptured GISTs [3].

We report herein a case of coexistence of gastric cancer and gastric GIST with intra-tumor bleeding managed successfully by TAE, which enabled radical resection after improving the patient’s condition. And we review the clinicopathological characteristics of simultaneous occurrence of gastric cancer and gastric GIST previously reported in Japanese literature.

## Case presentation

**Fig. 1:**
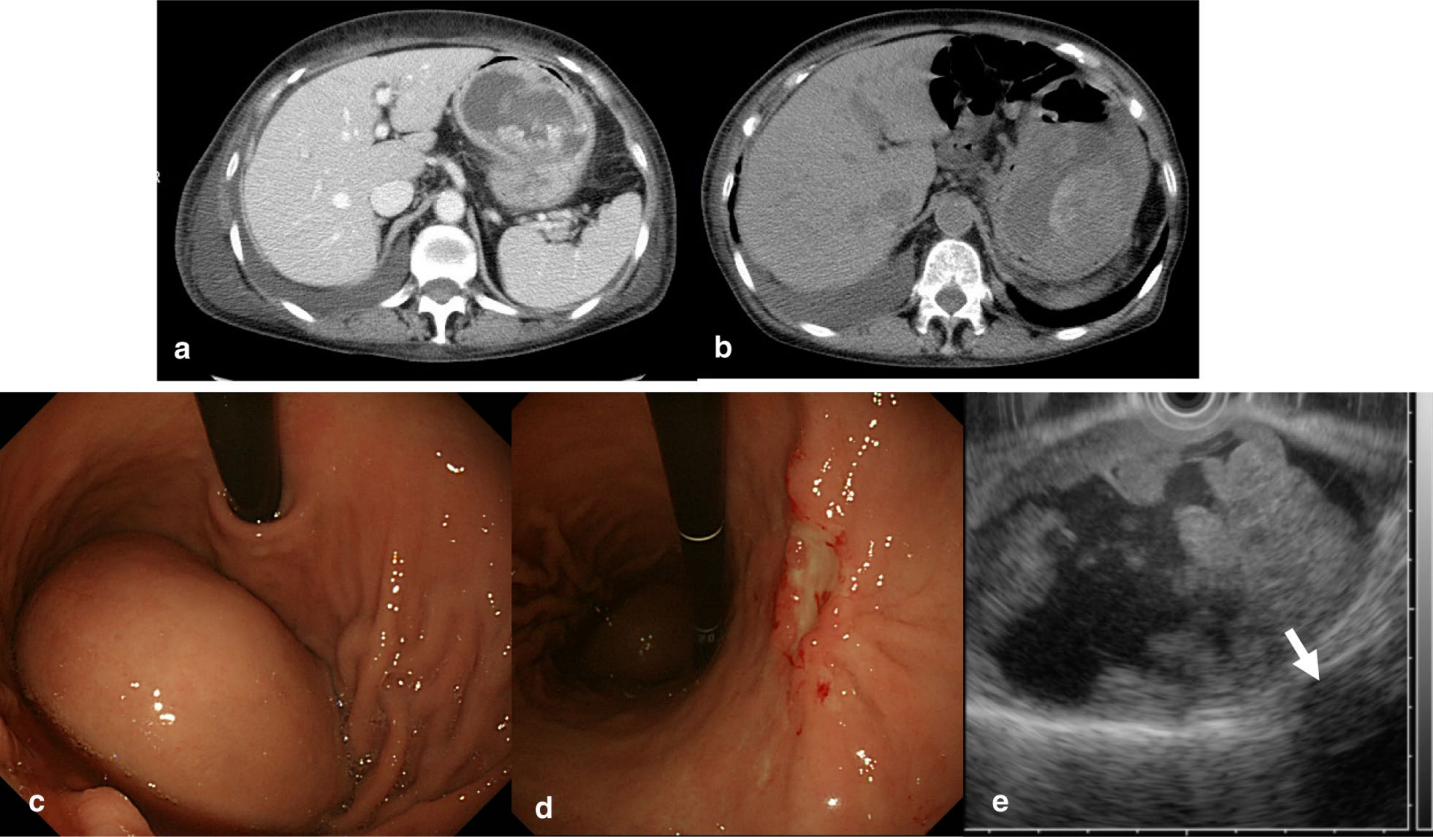
**a** On admission contrast-enhanced CT showed a mass of 60 × 80 mm in diameter at the fornix of the stomach, which had intra-tumor heterogeneity with enhanced margin. Intra-tumoral extravasation of contrast agent was observed. **b** Twelve days after admission plain CT showed that the tumor grew to 65 × 90 mm in diameter. **c** On endoscopy, a submucosal bulge was identified with overlying normal mucosa on the anterior wall at the fornix of the stomach. **d** A 0–IIc ulcerative lesion surrounded by abnormal edge of converging folds, namely club-like thickening, was perceived at the lesser curvature of the gastric angle. **e** Endoscopic ultrasound demonstrated that the subepithelial cystic tumor containing hypoechoic papillary component arising from the 4th layer of the gastric wall, muscularis propria (arrow)

A 75-year-old woman was referred to our hospital because of progressing dyspnea and systemic edema. Her previous history and family history were unremarkable. Laboratory tests revealed iron deficiency anemia with blood hemoglobin concentration of 6.1 g/dl and serum iron concentration of 8 μg/dl (reference value 48–154 μg/dl), but fecal occult blood was negative. Hypoproteinemia was observed with total protein of 4.0 g/dl (reference value 6.5–8.2 g/dl) and serum albumin of 2.2 g/dl (reference value 3.9–5.1 g/dl). Serum level of carcinoembryonic antigen (CEA) and carbohydrate antigen 19–9 (CA19-9) were within the normal range of 1.4 ng/ml and 7.3 U/ml, respectively. Chest radiograph showed cardiothoracic ratio of 70% with right costophrenic angle obliteration. Abdominal enhanced computed tomography (CT) demonstrated a tumor of 60 × 80 mm in diameter at the fornix of the stomach, which had intra-tumor heterogeneity with enhanced margin. Extravasation of contrast agent in the tumor was observed (Fig. 1a). Endoscopic examination showed a protuberant tumor covered with normal mucosa on the anterior wall of the fornix without any blood in the stomach (Fig. 1c). A type 0–IIc lesion surrounded by abnormal edge of converging folds, namely club-like thickening, was observed at the lesser curvature of the gastric angle (Fig. 1d). After transfusion of 4 units of red blood cell concentrate, her hemoglobin level rose to 9.5 g/dl.

**Fig. 2:**
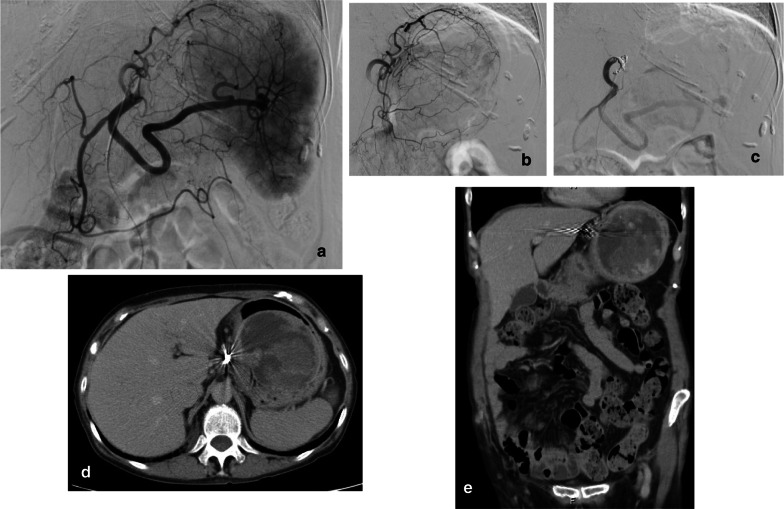
**a**, **b** Thirteen days after admission, angiogram of celiac artery and left gastric artery showed a hypovascular tumor surrounded by tumor vessels originated from the left gastric artery. Extravasation could not be detected. **c** Embolization with stainless steel coils and gelatin particles was performed successfully. Final angiogram showed relative stasis of the left gastric artery. **d**, **e** Axial and reconstructed coronal CT after TAE showed a large cystic tumor involving the gastric fundus and coils in the left gastric artery

For persistent anemia, additional 6 units of red blood cell concentrate were administered. Twelve days after admission, abdominal plain CT showed a submucosal tumor grew to 65 × 90 mm in diameter (Fig. 1b). Endoscopic ultrasound showed a subepithelial cystic tumor containing hypoechoic solid component with continuity to the 4th layer of the gastric wall as muscularis propria (Fig. 1e) [4]. Although boring biopsy was not performed to avoid tumor rupture, the patient was diagnosed as gastric GIST with intra-tumor bleeding. Tubular adenocarcinoma was detected in the coexisting 0–IIc lesion (T1b(sm)N0M0 cStageIB). Cytology of pleural effusion and ascites were negative for malignancy. Thirteen days after admission, TAE rather than emergency surgery was carried out because of her poor performance status of level 3. Angiography showed a hypovascular tumor surrounded by the tumor vessels originated from the left gastric artery at the fornix of the stomach (Fig. 2a, b). Embolization was performed with stainless steel coils and gelform particles were injected until relative stasis of the left gastric artery was achieved (Fig. 2c–e). After TAE, no blood transfusion was required throughout her hospital stay.

**Fig. 3:**
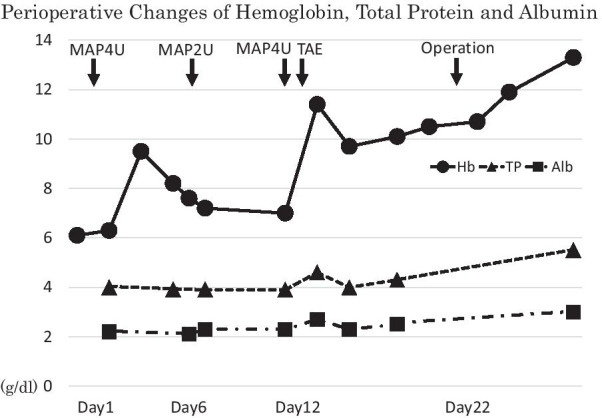
A chart of the perioperative course of hemoglobin, total protein and serum albumin levels showed that remission of anemia has been obtained after TAE. And elective surgery could be carried out

**Fig. 4:**
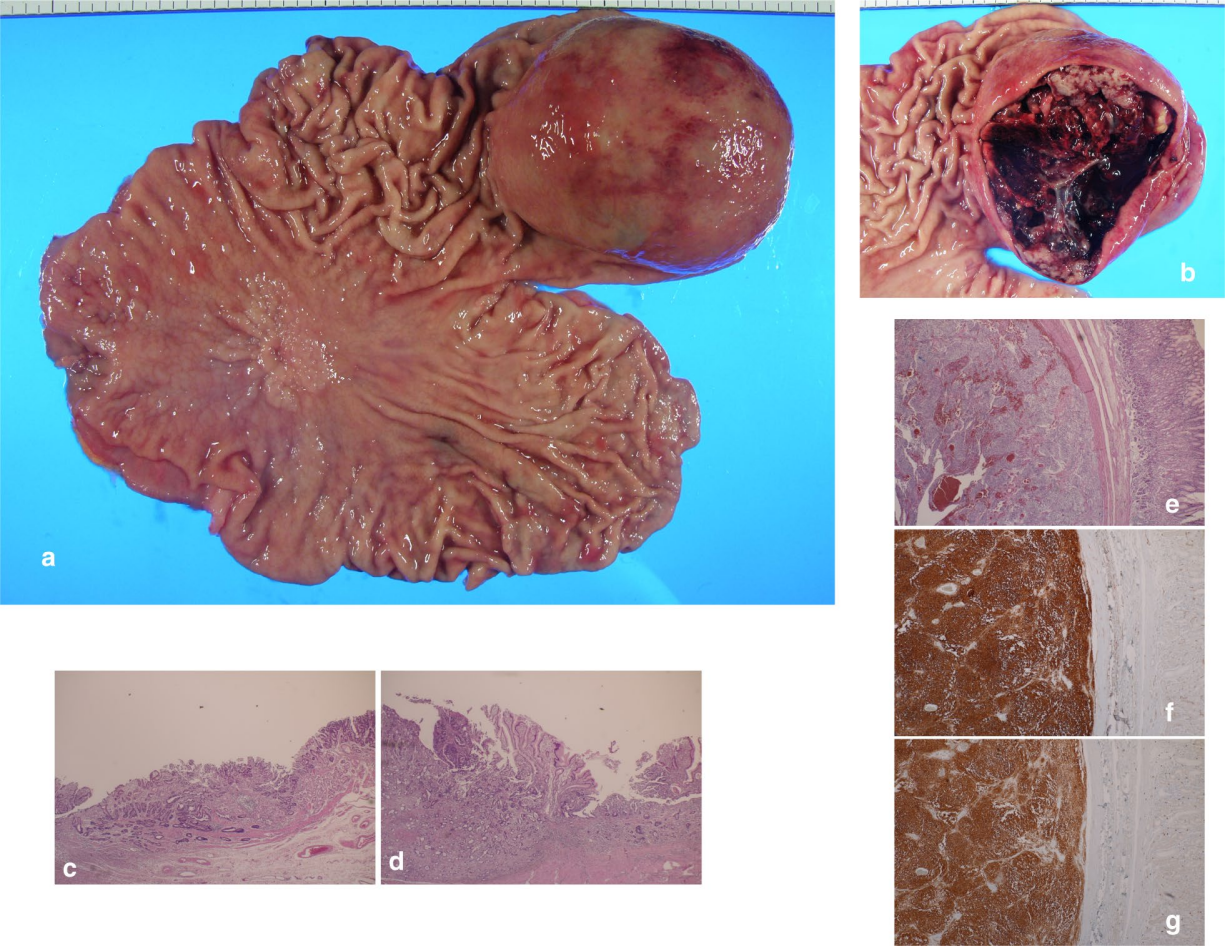
**a** Macroscopically total gastrectomy specimen showed a 0–IIc lesion measuring 34 × 50 mm and a submucosal tumor measuring 65 × 92 mm. **b** Contents of the cystic lesion were a papillary tumor constituting the cyst wall, necrotic tissue, and a large amount of coagula. **c**, **d** Histopathologically a 0–IIc lesion showed shallow infiltration to muscularis propria by well-differentiated adenocarcinoma (hematoxylin and eosin stain, magnification ×20). **e** Papillary tumor was composed of spindle-shaped cells with oval nuclei (hematoxylin and eosin stain, magnification ×20). **f**, **g** Immunohistochemical studies indicated strong staining for CD34 (**f**) and CD117(c-KIT) (**g**) (magnification ×20)

Nine days after TAE, open total gastrectomy with D1-lymph node dissection was conducted (Fig. 3) to avoid intraoperative rupture of bulky GIST. Neither peritoneal dissemination nor liver metastasis was observed. Macroscopically resected specimen showed a 0–IIc lesion measuring 34 × 50 mm and a submucosal tumor measuring 65 × 92 mm at the stomach (Fig. 4a, b). Histopathologically the 0–IIc lesion showed shallow infiltration to the proper muscle by well-differentiated adenocarcinoma (Fig. 4c, d). There was slight vascular invasion and 1 out of 44 lymph node metastasis was observed (pT2N1M0 fStageIIA). The submucosal nodule was consistent with GIST of the intermediate-risk category [5], which was composed of spindle-shaped cells with oval nuclei and showed a low mitotic index (< 5/50HPF) (Fig. 4e). The immunohistochemical studies indicated strong staining for CD34 and CD117(c-KIT) (Fig. 4f, g). These findings confirmed simultaneous development of gastric cancer and gastric GIST. The postoperative course was uneventful. After providing informed consent, the patient did not accept postoperative adjuvant therapy with S1 or imatinib mesylate and discharged 22 days after surgery.

## Discussion

GIST is the most common mesenchymal tumor [6], and located in stomach (60–70%) followed by small bowel (20–30%), colon–rectum (5%-10%) and esophagus (5%) [7], but account for only 2% of all malignant gastric tumors [8, 9]. GIST is classified into 4 risk groups with reference to tumor size, mitotic count [5], location and presence of tumor rupture [10, 11].

The incidence of another neoplasm with GIST was reported in the range of 17.1 to 37.9% [12–14]. Synchronous occurrence of gastric GIST and gastric cancer has been reported more frequently in recent years. According to the review of 4813 symptomatic GIST, the frequency of coexisting malignant tumor was 10.1% and that of gastric cancer was about 2% [10]. The Japanese study of 109 GIST cases reported that 21.3% had malignant tumors and only 1 case (0.9%) had gastric cancer [15]. Because the prevalence of gastric cancer is 102/1,00,000/year [16] in Japan, the coexistence rate of gastric cancer with gastric GIST in 0.9–2% may mean that it occurs more frequently than coincidence. It was documented that the incidence of gastric GIST in 2035 gastric cancer cases was 0.29% [17] and that in 560 Japanese cases was 1.4% [18]. The coexistence rate is much higher than the prevalence of GIST (1–2/100000/year) [1], because it contains microscopic GISTs in the resected specimens for gastric cancer. It is hypothesized that there are possible associations between gastric GIST and gastric cancer; however, no data are available. It was reported that GIST was found in 0.2% of autopsy and microscopic GISTs (less than 10 mm in diameter) [13] coexisted with 35% of resected specimens of gastric cancer [19]. Considering the existence of latent GISTs, the prevalence of the synchronous occurrence of gastric cancer and gastric GIST may be more common.

The 40 cases, including our case, of simultaneous development of gastric cancer and gastric GIST have been reported to date in Japan (Table1) [20, 21]. In summary, the male–female ratio exhibited 34:6 with strong male predominance, even though there are no gender differences in GIST and approximately double in gastric cancer. Half of GISTs were latent, which were diagnosed during or after surgery for gastric cancer. In case of GISTs diagnosed before surgery, 10 were symptomatic and another 10 were incidental. Only 15% (6/40) were histologically diagnosed as GIST before surgery. The more aggressively endoscopic ultrasound fine-needle aspiration will be conducted, the more preoperative diagnosis will increase. When the reported cases were divided by the size of GIST at 20 mm in diameter, they were divided into 20 cases each (Table 2). GISTs of less than 20 mm in diameter belonged to very low risk group in Fletcher classification [5], and all were latent GISTs. Among the incidental GISTs, 7 patients were diagnosed at the same time of gastric cancer and 3 were diagnosed during preoperative inspection of gastric cancer. Thirteen patients who had symptoms of GIST and/or gastric cancer exhibited bleeding from GIST in 7 (hematochezia in 3, hematemesis in 2 and anemia in 2), fundic obstruction in 1 and unspecific symptoms in 5 (fullness in 2 and abdominal pain in 3). It was reported that the prognosis of coexistence of gastric cancer and gastric GIST depended primarily upon the progression of gastric cancer [10, 11]. The rupture of GIST was associated with grave prognosis [19]. Because the occurrence of these conditions is uncommon, it is unknown which affects their prognosis. Since 17% of rupture of GIST occurred during laparotomy or laparoscopy [2], a meticulous care to avoid intraoperative rupture is mandatory.

**Table 1:** Summary of synchronous gastric cancer and GISTs of the stomach in Japanese literature (*n* = 40)

Age	M/F	GIST				
		Occupied region U/M/L	Size (mm)	Fletcher classification H:I:L:vL	Pre-op. diag. (pathological)	Region match (collision)
72.0 ± 6.66	34/6	17/17/6	37.8 ± 31.9	5:6:7:18	20 (6)	10 (6)

Order of diagnosis in pre-op. diag. GIST cases (*n* = 20)	Symptoms of pre-op. diag. GIST cases (*n* = 13 * + **)	Gastric cancer			
		Occupied region U/M/L	Size (mm)	Stage	Operation procedures
GIST → GCa 10* GCa → GIST 3** GIST = GCa 7*** (via Medical check-up) (**,***incidental GIST)	Hematochezia 3 Hematemesis 2 Anemia 2 Flatulence 2 Epigastralgia 3 Obstruction 1	15/13/14	38.0 ± 30.1	IA21 IB3 IIA5 IIB2 IIIA1 IIIB2 IV4	Total gastrectomy 19 Proximal gastrectomy 1 Distal gastrectomy 16 Local resection 3 Completion gastrectomy 1

**Table 2:** Summary of synchronous gastric cancer and GISTs of the stomach in Japanese literature (*n* = 40)

Size of GIST	Age	M/F	Location U/M/L	Size of GIST (cancer) (mm)	Fletcher classification H:I:L:vL	Pre-op. diag	Location match
20 mm≦ (*n* = 20)	70.6 ± 6.77	17/3	9/8/3 (7/6/7)	67.6 ± 29.1 (37.2 ± 20.7)	5:6:7:0	20	2
20 mm > (*n* = 20)	73.4 ± 6.71	17/3	8/9/3 (8/6/6)	8.09 ± 2.62 (38.8 ± 19.5)	0:0:0:18	0	8

Eighty percent of ruptured GISTs developed in small bowel and 11% in stomach [2]. More than 5 cm in diameter has been reported as one of the risk factors for rupture [22]. Ruptured gastric GIST can usually present with occult bleeding, so a life-threatening bleeding is rare at 1% [23]. In case of 1241 symptomatic gastric GISTs, 626 (54%) patients presented with intra-luminal and 21 (1.7%) with intra-peritoneal bleeding [24]. The frequency of intra-tumor bleeding, presumed to be in a pre-rupture state, is unclear. Although rupture of gastric GIST might be difficult for endoscopic management, TAE is widely accepted for the treatment of gastroduodenal bleeding resistant to medical and endoscopic therapy especially in elderly patients at risk for postoperative morbidity [25]. If operation can be avoided, mortality is halved [26]. There have been only a few reports regarding TAE for gastrointestinal bleeding from GIST [3, 27, 28]. Twenty (1.2%) patients of 2078 pathologically proven GIST underwent TAE for gastrointestinal bleeding with technical success rate of 95% and clinical success rate of 90% [3].

In the present case, we opted to TAE for the gastric GIST before rupture effectively. TAE for GIST, with or without rupture, might facilitate induction of the tumor necrosis and decrease the tumor burden, which would subsequently allow minimal invasive surgery [27–29].

## Conclusions

Simultaneous occurrence of gastric cancer and gastric GIST was speculated to be more common. Coexisting GISTs were usually small and detected incidentally during or after surgery. Surgeons and pathologists should be alert to perform thorough perioperative investigation. TAE for ruptured GIST may be effective for hemostasis and reduction of tumor burden, which would facilitate minimal invasive surgery.

## Data Availability

All the data in this article are available from the corresponding author upon reasonable request.

## Abbreviations

GIST: Gastrointestinal stromal tumor
TAE: Transcatheter arterial embolization
